# Improving inhaler adherence in patients with Chronic Obstructive Pulmonary Disease: a cost-effectiveness analysis

**DOI:** 10.1186/1465-9921-15-66

**Published:** 2014-06-14

**Authors:** Job FM van Boven, Eline Tommelein, Koen Boussery, Els Mehuys, Stefan Vegter, Guy GO Brusselle, Maureen PMH Rutten-van Mölken, Maarten J Postma

**Affiliations:** 1 Department of Pharmacy, Unit of PharmacoEpidemiology & PharmacoEconomics, University of Groningen, Antonius Deusinglaan 1, 9713 AV Groningen, The Netherlands; 2 Pharmaceutical Care Unit, Faculty of Pharmaceutical Sciences, Ghent University, Harelbekestraat 72, 9000 Ghent, Belgium; 3 Department of Respiratory Medicine, Ghent University Hospital, De Pintelaan 85, 9000 Ghent, Belgium; 4 Departments of Epidemiology and Respiratory Medicine, Erasmus Medical Center, Rotterdam, The Netherlands; 5 Department of Health Economics (iMTA), Institute for Medical Technology Assessment, Erasmus University, J-building - Campus Woudestein, PO Box 1738, Rotterdam, The Netherlands

**Keywords:** Adherence, Cost-effectiveness, Inhalation technique, Intervention, Pharmacist

## Abstract

**Background:**

The PHARMACOP-intervention significantly improved medication adherence and inhalation technique for patients with COPD compared with usual care. This study aimed to evaluate its cost-effectiveness.

**Methods:**

An economic analysis was performed from the Belgian healthcare payer’s perspective. A Markov model was constructed in which a representative group of patients with COPD (mean age of 70 years, 66% male, 43% current smokers and mean Forced Expiratory Volume in 1 second of % predicted of 50), was followed for either receiving the 3-month PHARMACOP-intervention or usual care. Three types of costs were calculated: intervention costs, medication costs and exacerbation costs. Outcome measures included the number of hospital-treated exacerbations, cost per prevented hospital-treated exacerbation and cost per Quality Adjusted Life-Year. Follow-up was 1 year in the basecase analysis. Sensitivity and scenario analyses (including long-term follow-up) were performed to assess uncertainty.

**Results:**

In the basecase analysis, the average overall costs per patient for the PHARMACOP-intervention and usual care were €2,221 and €2,448, respectively within the 1-year time horizon. This reflects cost savings of €227 for the PHARMACOP-intervention. The PHARMACOP-intervention resulted in the prevention of 0.07 hospital-treated exacerbations per patient (0.177 for PHARMACOP versus 0.244 for usual care). Results showed robust cost-savings in various sensitivity analyses.

**Conclusions:**

Optimization of current pharmacotherapy (e.g. close monitoring of inhalation technique and medication adherence) has been shown to be cost-saving and should be considered before adding new therapies.

## Background

Chronic Obstructive Pulmonary Disease (COPD) involves a high burden on morbidity, mortality as well as healthcare and societal costs [1,2]. In Belgium, between 5.5% (population aged 55 years) and 9.5% (population aged 75 years) of the population is suffering from COPD [3]. Although COPD is known by its progressive character, disease symptoms can be well managed through proper medications and optimal disease management. For this purpose, the Global Initiative for Chronic Obstructive Lung Disease (GOLD) guidelines recommend close monitoring of patients’ pharmacotherapy, including medication adherence and inhalation technique [4]. Both adherence and inhalation techniques have been shown to be suboptimal in patients with COPD [5]. Moreover, suboptimal adherence and inhalation mishandling are significantly associated with worsened clinical, humanistic and economic outcomes [6,7].

Several intervention programs have been developed to improve disease management, of which multidisciplinary collaborations to provide integrated care have been shown to be particularly effective [8]. In recent years, community pharmacists are being increasingly involved in COPD management [5,9,10]. Due to their periodical patient contacts upon prescription refills and their specific knowledge on the (inter)acting and administration of medication, community pharmacies offer a promising platform for optimization of medication adherence and inhalation techniques of patients with COPD.

The 3-month PHARMACOP-intervention (PHARMAceutical Care for COPD, N = 734), conducted in 170 community pharmacies in Belgium, significantly improved both medication adherence and patients’ inhalation technique [11]. In addition, significantly lower hospitalization rates were observed in the intervention group as compared to the usual care group. In times of increasing healthcare costs and higher demands, economic analyses of healthcare interventions are becoming of increasing importance to achieve a fair allocation of scarce healthcare resources. Cost-effectiveness of several COPD disease management programs have been studied before [12,13]. A recent meta-analysis showed that such programs can lead to significant savings in hospital costs and total healthcare costs [14]. However, economic analyses of COPD programs primarily focusing on medication adherence and inhalation technique are limited and therefore recommended [15]. This study aimed to assess the cost-effectiveness of the PHARMACOP community pharmacists’ COPD intervention program.

## Methods

We performed a cost effectiveness analysis of the PHARMACOP-study [11]. Details of the original randomized controlled trial and the methods related to the cost-effectiveness analysis are described in the following sub-sections.

### PHARMACOP-study

The PHARMACOP-study was a 3-month randomized controlled trial (N = 734) carried out between December 2010 and July 2011 in 170 community pharmacies throughout Belgium. Interventions focused on improving medication adherence and inhalation technique. Results showed that inhalation scores were significantly improved with 13.5% (95%CI: 10.8-16.1; P < 0.0001). Medication adherence, as measured by the medication refill adherence (MRA) [16], was significantly improved from 85.70% to 94.21% (difference: 8.51%, 95%CI: 4.63-12.4; P < 0.0001). In the intervention group a significantly lower hospitalization rate was observed (9 vs 35; Rate ratio: 0.28, 95%CI: 0.12-0.64; P = 0.003). No other significant differences were observed. A summary of the PHARMACOP-study is provided in Additional file 1 and the complete description of the study protocol and its results can be found elsewhere [11].

### Cost effectiveness analysis

The cost-effectiveness analysis was performed according to the Belgian guidelines for pharmacoeconomic research. The PHARMACOP-trial followed patients for up to 3 months. However, as some costs and effects resulting from the intervention are expected to occur after this period, a Markov model was constructed to be able to capture long-term costs and effects of the PHARMACOP-intervention. Markov models have often been used in health economic evaluations of COPD interventions [17,18] and are a recommended approach to increase external validity and to allow for long-term follow-up [19,20]. Details of the model are described in the following part.

### Study perspective

The analysis was performed from a healthcare payer’s perspective, in line with the recommendations from the Belgian guidelines for pharmacoeconomic research [21]. This means that the analysis included only direct healthcare costs, such as primary care, hospital care and medications; i.e. public payments as well as co-payments by the patient (in Belgium known as “remgeld”). No indirect costs (of productivity losses) were included.

### Comparison

**Figure 1:**
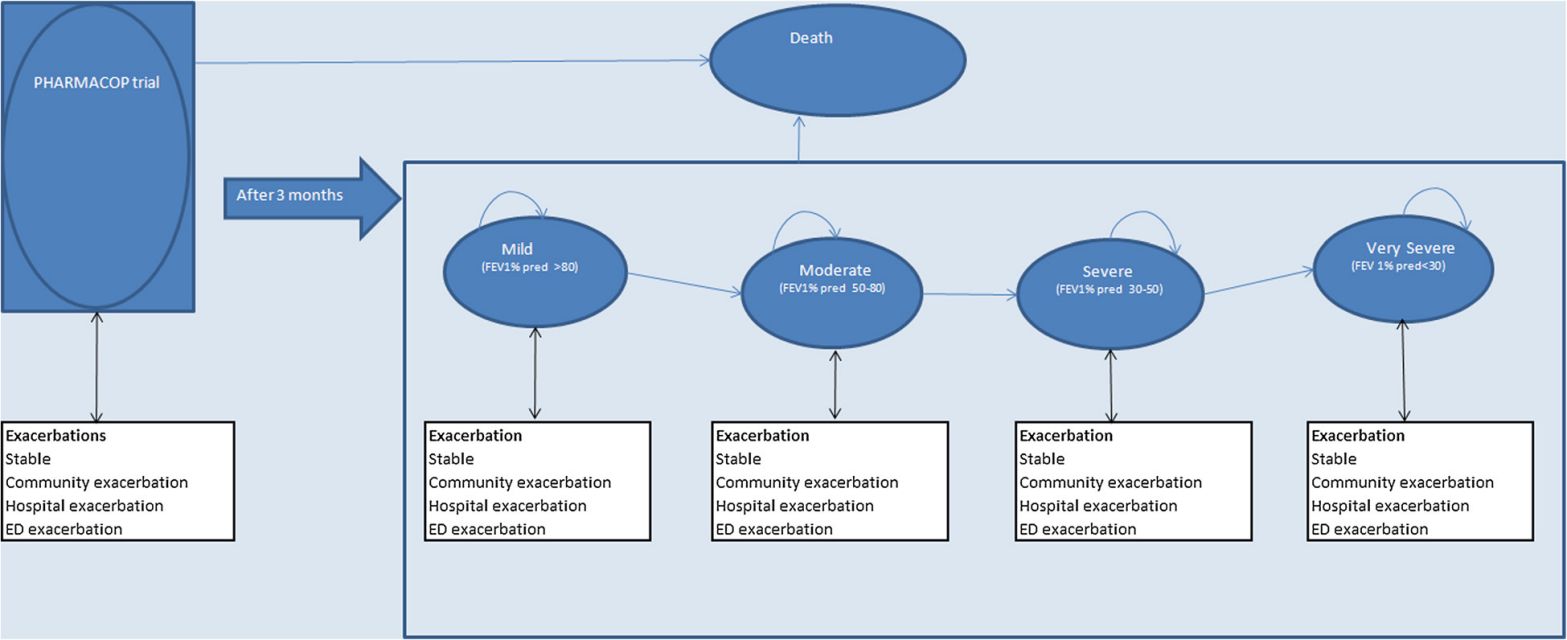
**Markov model to follow patients with COPD in time.** ED: Emergency Department; FEV1% pred: percentage of the predicted Forced Expiratory Volume in 1 second.

In the model (Figure 1), a hypothetical group of patients with COPD was followed either receiving the PHARMACOP-intervention or usual care. The model population, with a start age of 70 years, 66% male and 43% current smokers, was an accurate reflection of the population participating in the PHARMACOP-trial [11]. Moreover, the community pharmacy population is considered representative for the COPD population in Belgium as all patients, disregarding insurance or disease severity, are refilling their prescriptions in community pharmacies.

Because the trial did not collect any measures related to patients’ spirometric functions we assumed a truncated (at 0 and 100%) normal distribution of Forced Expiratory Volume in 1 second (FEV1% predicted) with a mean of 50% and a standard deviation of 19.9 to obtain a severity distribution with 16% of patients in the very severe, 34% in severe, 43% in moderate and 7% in mild COPD state. This assumption was based on characteristics from the PHARMACOP-study: Patients were included in the PHARMACOP-study if they used at least one type of long-acting drug, indicating a disease state worse than GOLD 1 [3]. In addition, mean COPD Assessment Test (CAT) score of patients in the PHARMACOP-trial was 16.5 (see Additional file 1), indicating marked symptoms [3]. The impact of this arbitrary mean percentage of FEV_1_% predicted was investigated in sensitivity analyses.

### Model structure

A Markov model was constructed in Microsoft® Excel 2010. In line with the length of the PHARMACOP-trial, the cycle length of the model was 3 months. In the first 3 months, patients started in the ‘PHARMACOP phase’ in which trial effects were directly projected at the model population. For validation purposes, after running the first cycle, results were compared to the results of the PHARMACOP-trial (Additional file 2). In the follow-up of the model (after the first cycle of three months), patients could move between five disease states: mild, moderate, severe, very severe and death (Figure 1) in line with the GOLD spirometric classifications [4].

### Model parameters

Model input and rationale are described in the following section and summarized in Table 1.

**Table 1:** Input parameters used in the model

**Parameter**	**Value**	**95%CI**	**Distribution**	**Source**
*Exacerbation rate (in first 3 months)*				
Community treated	0.534	0.483-0.586	Normal	[11]
ED treated	0.050	0.028-0.072	Poisson	[11]
Hospital treated	0.096	0.063-0.129	Poisson	[11]
*Intervention effects trial*				
RR community treated exacerbation	0.903	0.737-1.106	LogNormal	[11]
RR ED treated exacerbation	0.815	0.411-1.618	LogNormal	[11]
RR hospital treated exacerbation	0.252	0.121-0.523	LogNormal	[11]
Medication adherence improvement (%)	8.51	4.62-12.40	Normal	[11]
*Exacerbation rate (>3 months, per year)*				
Mild state - Community treated	0.61	0.34-1.10	LogNormal	Derived
Mild state - ED treated	0.06	0.01-0.30	LogNormal	Derived
Mild state - Hospital treated	0.11	0.02-0.56	LogNormal	[22]
Moderate state - Community treated	0.89	0.70-1.12	LogNormal	Derived
Moderate state - ED treated	0.08	0.04-0.18	LogNormal	Derived
Moderate state - Hospital treated	0.16	0.07-0.33	LogNormal	[22]
Severe state - Community treated	1.22	1.14-1.31	LogNormal	Derived
Severe state - ED treated	0.11	0.11-0.12	LogNormal	Derived
Severe state - Hospital treated	0.22	0.20-0.23	LogNormal	[22]
Very severe state - Community treated	1.55	1.11-2.17	LogNormal	Derived
Very severe state - ED treated	0.14	0.07-0.30	LogNormal	Derived
Very severe state - Hospital treated	0.28	0.14-0.63	LogNormal	[22]
*Costs (€)*				
Intervention fixed	10,000	8,136-12,053	Gamma	Estimate
Intervention per-patient per 3 months	50	29-77	Gamma	Estimate
Medication (100% adherence) per year	1,022	790-1285	Logistic	[23]
Exacerbation community treated	106	60-163	Gamma	[24]
Exacerbation ED treated	712	407-1100	Gamma	[24]
Exacerbation hospital treated	5,617	5,557-5,677	Gamma	[25]
*Utilities*				
Mild COPD state	0.897	0.8561-0.9319	Beta	[26]
Moderate COPD state	0.755	0.6921-0.8131	Beta	[26]
Severe COPD state	0.748	0.6761-0.8138	Beta	[26]
Very severe COPD state	0.549	0.4325-0.6634	Beta	[26]
Exacerbation community treated	-0.0166	0.0126-0.0212	Beta	[27]
Exacerbation ED treated	-0.0300	0.0244-0.0361	Beta	Estimate
Exacerbation hospital treated	-0.0482	0.0326-0.0666	Beta	[27]

*CI*: Confidence Interval; *€*: 2013 euros; *ED*: Emergency Department; *RR*: Relative Risk.

#### *COPD disease progression*

Transition between disease states was based on the annual decline in the mean FEV_1_% predicted. In each cycle, a basic decline in FEV_1_% predicted was modeled depending on age, gender and smoking status. The annual decline was based on a previously published regression model [28] that was fitted to longitudinal data from the Lung Health Study [29]. We assumed that the PHARMACOP-intervention did not affect decline in FEV_1_% predicted. The PHARMACOP-trial did not report any effects on mortality. Therefore, in order to obtain estimates of the size of the COPD cohort in time, Belgian age-depended COPD and all-cause mortality was modeled and no effects of the PHARMACOP-intervention were assumed [3]. Mortality due to exacerbations was not modeled.

#### *Exacerbations*

During each cycle, patients had a chance of three different kinds of exacerbations to occur: those that were community-treated only, those that led to an Emergency Department (ED) visit and those that led to hospitalization. In the control group, exacerbation rates from the PHARMACOP control group were applied in the first three months (Table 1) and after three months exacerbation rates from a meta-analysis were applied [22]. In the intervention group, these exacerbation rates were multiplied by the effects (relative risks; RR) from the PHARMACOP-intervention on all three types of exacerbations (Table 1). In the basecase this effect lasted only for the first three months, in line with the follow-up of PHARMACOP. In sensitivity analyses, effects that lasted for 1, 5 and 12.5 years were also analyzed. In these long-term projections linear extrapolation of the intervention effects was applied.

#### *Costs*

All costs were expressed in 2013 euros. Three types of costs were calculated: intervention costs, medication costs and exacerbation costs. Intervention costs included a fixed initial fee for training of the pharmacists and written materials (estimated at €10,000) and a per-patient fee. The fixed intervention costs were divided by 363 patients to obtain the costs per patient. The per-patient fee was based on the average time investment for the two face-to-face counseling sessions (mean total time: 38 minutes, SD: 21 minutes) provided in the 3-months of the PHARMACOP-trial, multiplied by the pharmacist’s salary, resulting in an estimated per-patient fee of approximately €50 (including employers premiums) per 3 months. Medication costs in the usual care group and the intervention group were calculated as the yearly costs of medications used in the PHARMACOP-trial when all medication would be used as prescribed (=100% adherence), multiplied by the actual ‘usual care group’ adherence (85.70%) or the ‘intervention group’ adherence (94.21%), respectively. Exacerbation costs were calculated as the product of the number of exacerbations (community, ED or hospital-treated) and the price per unit (Table 1).

#### *Utilities*

Each COPD disease state was assigned a preference-based health-related quality of life value (a utility) [26]. In addition, a percentage of utility decrement from baseline was modeled if an exacerbation occurred (Table 1). Notably, the PHARMACOP-intervention showed no significant effects on quality of life as measured by the EQ-5D, a result that might be related to the timing of the pre-scheduled pharmacy-visits for measurement of health status and the relatively short duration of exacerbations [20]. Therefore, as an conservative approach, no direct effects on quality of life were applied in the intervention group and utility values were solely based on disease state and the occurrence of exacerbations. Consequently, Quality Adjusted Life Years (QALY) gains due to the PHARMACOP-intervention only result from a reduction in exacerbations.

### Time horizon

The time horizon in the basecase analysis was 1 year. This time horizon was chosen to align with budgetary timeframes from (Belgian) health insurance companies. However, as effects from the interventions may impact on the long-term, costs and effects using longer-term follow-up (up to 12.5 years) were assessed in sensitivity analyses. Effects on medication adherence (and related medication costs) were assumed to last for 1 year after the program ended, based on delayed effects on adherence shown in previous studies [30]. After 1 year, adherence went back to baseline adherence (85.7%).

### Outcomes

The model calculated cost per QALY gained and cost per hospital-treated exacerbation avoided. The generic outcome (cost per QALY) was reported to enable comparisons of cost-effectiveness across disease areas. The COPD specific outcome (hospital-treated exacerbations) was included to compare this intervention across the field of COPD interventions. The incremental cost-effectiveness ratio (ICER) was calculated as:

$$\begin{array}{c} \mathrm{ICER}=\left[{\text{Costs}}_{\text{PHARMACOP}}–{\text{Costs}}_{\text{usual}\;\text{care}}\right]/\\ \;\left[{\text{Effect}}_{\text{PHARMACOP}}–{\text{Effect}}_{\text{usual}\;\text{care}}\right]\\ \;=\Delta C/\Delta E \end{array}$$

### Sensitivity & scenario analyses

To address parameter, structural and methodological uncertainty, both univariate and probabilistic sensitivity analyses were performed. To show individual influence of the parameters, all relevant parameters were varied within their 95% confidence intervals (95%CI) and outcomes were presented in a tornado diagram showing the most influential parameters on top of the graph. In probabilistic sensitivity analyses (3,000 iterations, using Monte Carlo simulations), all relevant parameters were varied primarily based on pre-specified statistic distributions as shown in Table 1[31]. The distribution for medication costs was fitted (best fit selected using Akaike Information Criterion) as patient-level data were available. Results of the probabilistic sensitivity analyses were presented in a cost-effectiveness plane. In scenario analyses the influence of different time horizons, long lasting effects of improved adherence and different program runtimes on cost-effectiveness were assessed. Sensitivity analyses were performed for alternative follow-up periods of respectively 0.5 year, 2, 5, 10 and 12.5 years. In long-term follow-up, all future costs and benefits (after 1 year) were discounted according to the Belgian pharmacoeconomic guidelines; costs at a rate of 3.0% and effects at a rate of 1.5% [21].

## Results

### Cost-effectiveness

**Figure 2:**
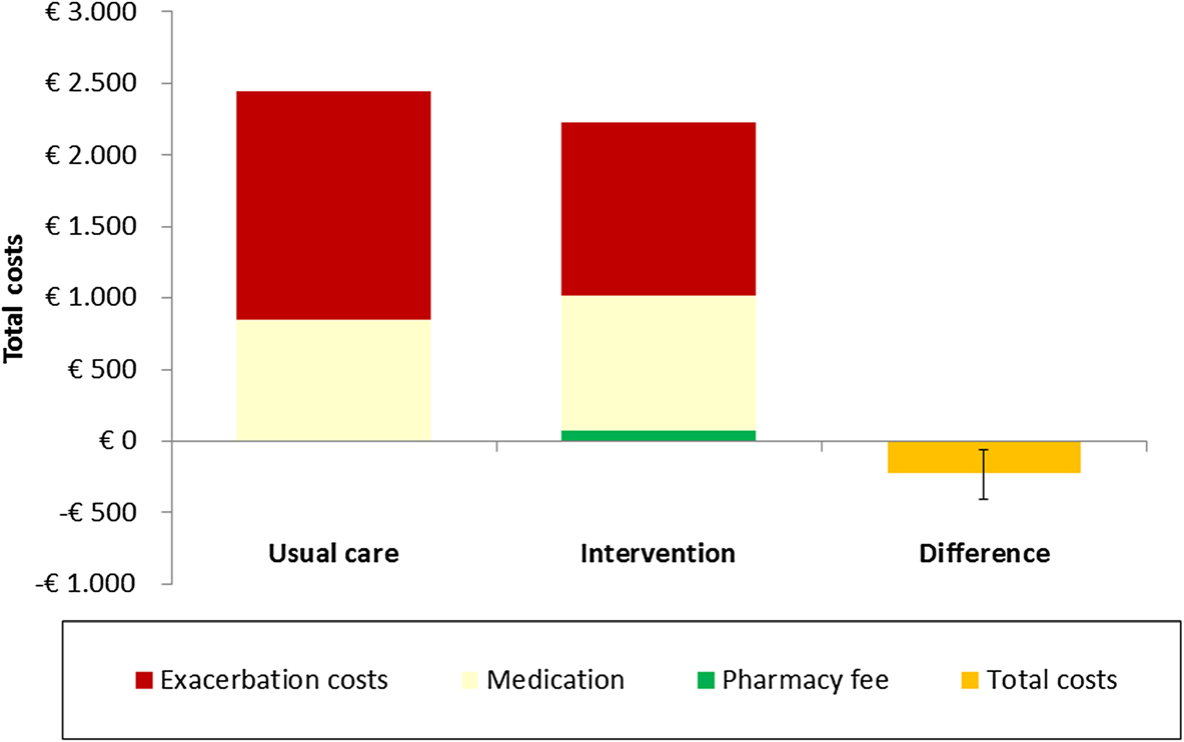
**Summary of 1-year effects on costs.***Usual care:* Medication costs (850), Pharmacy fee (0), Exacerbation costs (1598), Total costs (2448); *Intervention:* Medication costs (934), Pharmacy fee (77), Exacerbation costs (1210), Total costs (2221); *Difference (95% CI):* Medication costs (84; 44-129), Pharmacy fee (77; 55-104), Exacerbation costs (-388; -225 - -560), Total costs (-227; -58 - -403).

**Figure 3:**
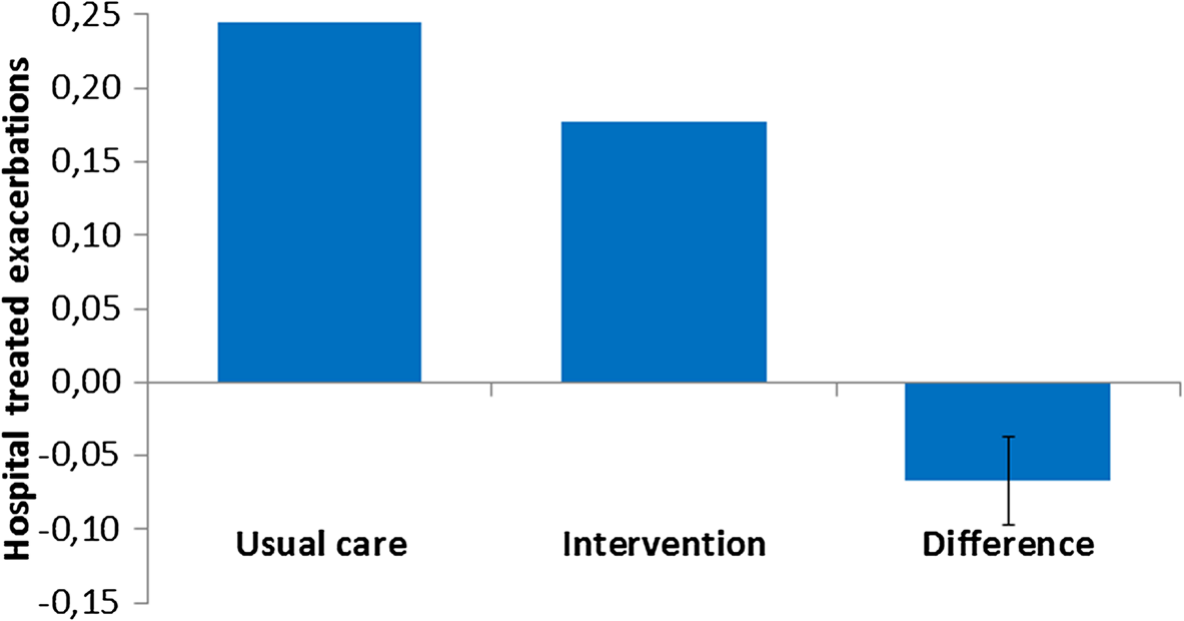
**Summary of 1-year effects on hospital-treated exacerbations.***Usual care:* Hospital Treated (HT) Exacerbations (0.24). *Intervention:* Hospital Treated (HT) Exacerbations (0.18). *Difference (95% CI):* Hospital Treated (HT) Exacerbations (-0.07; -0.04 - -0.10).

The total costs per patient for intervention and usual care were €2,221 and €2,448, respectively within the 1-year time horizon in the basecase (Figure 2). This reflects a cost saving of €227 (95%CI: €58-€403) per patient for the PHARMACOP-intervention. Also, the PHARMACOP-intervention resulted in a significant decrease of 0.07 (95%CI: 0.04-0.10) hospital-treated exacerbations per patient (0.177 for PHARMACOP versus 0.244 for usual care) when the intervention effect was applied for the first 3-months (Figure 3). In addition, a small (<0.001 QALYs) increase in QALYs gain was observed. Notably, the initial higher costs in the PHARMACOP-intervention (due to intervention costs and increased adherence) compared to usual care of €161 per patient were offset by €388 savings on expenses for treatment of exacerbations.

### Sensitivity analyses

**Figure 4:**
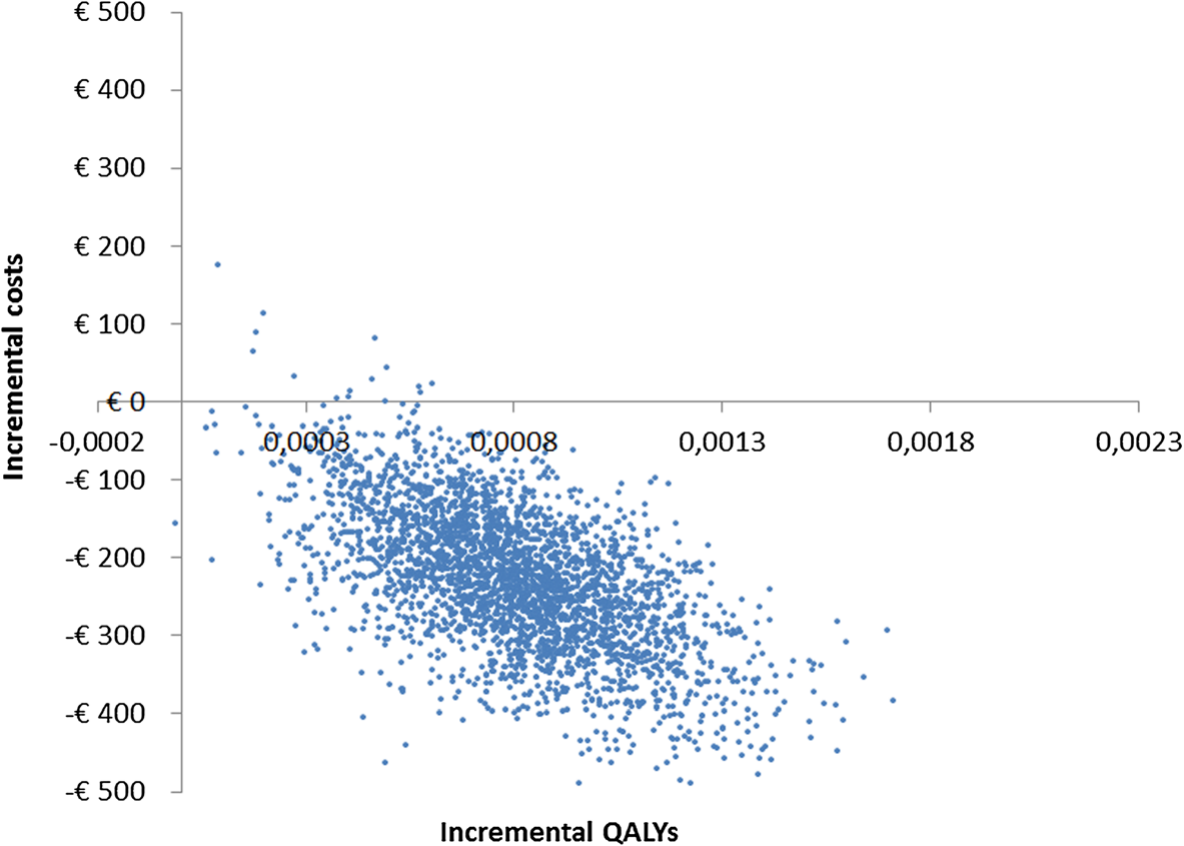
**Probabilistic sensitivity analyses for QALYs.** QALY: Quality Adjusted Life-Year.

**Figure 5:**
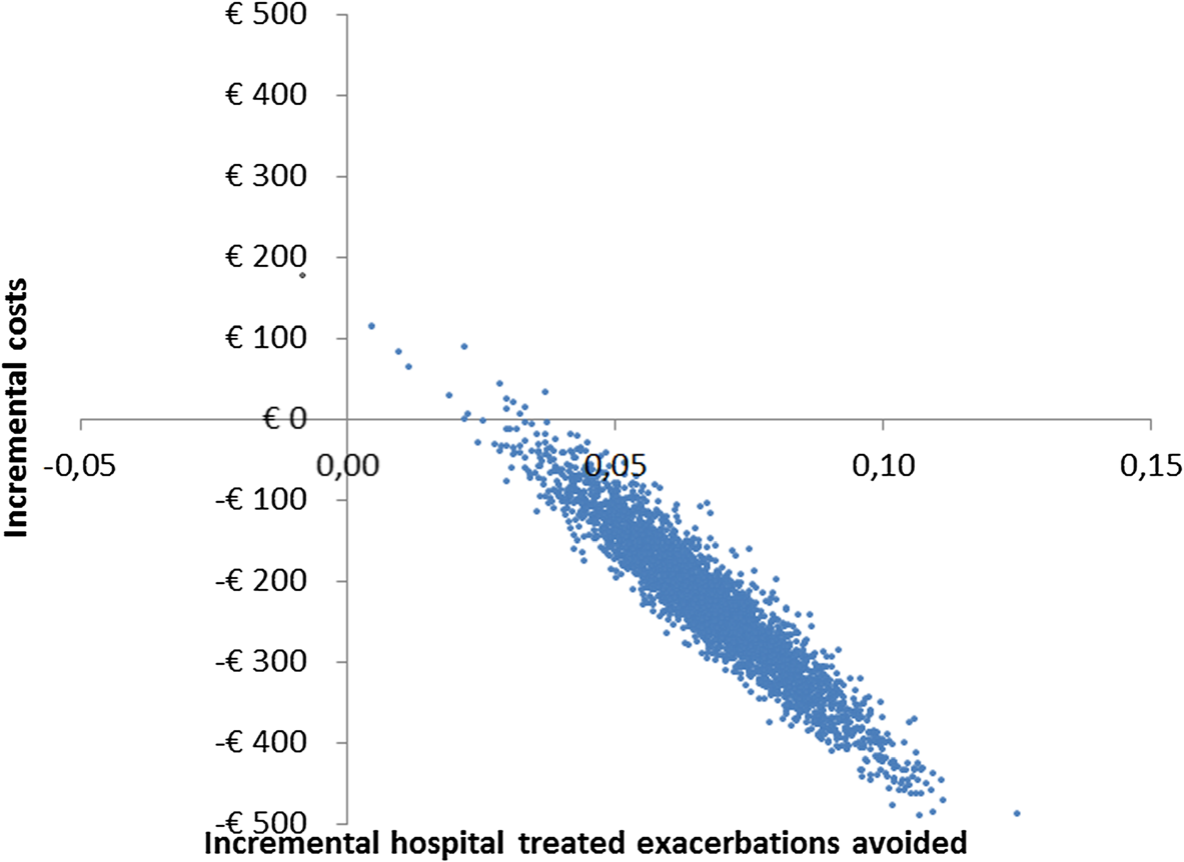
Probabilistic sensitivity analyses for hospital-treated exacerbations.

**Figure 6:**
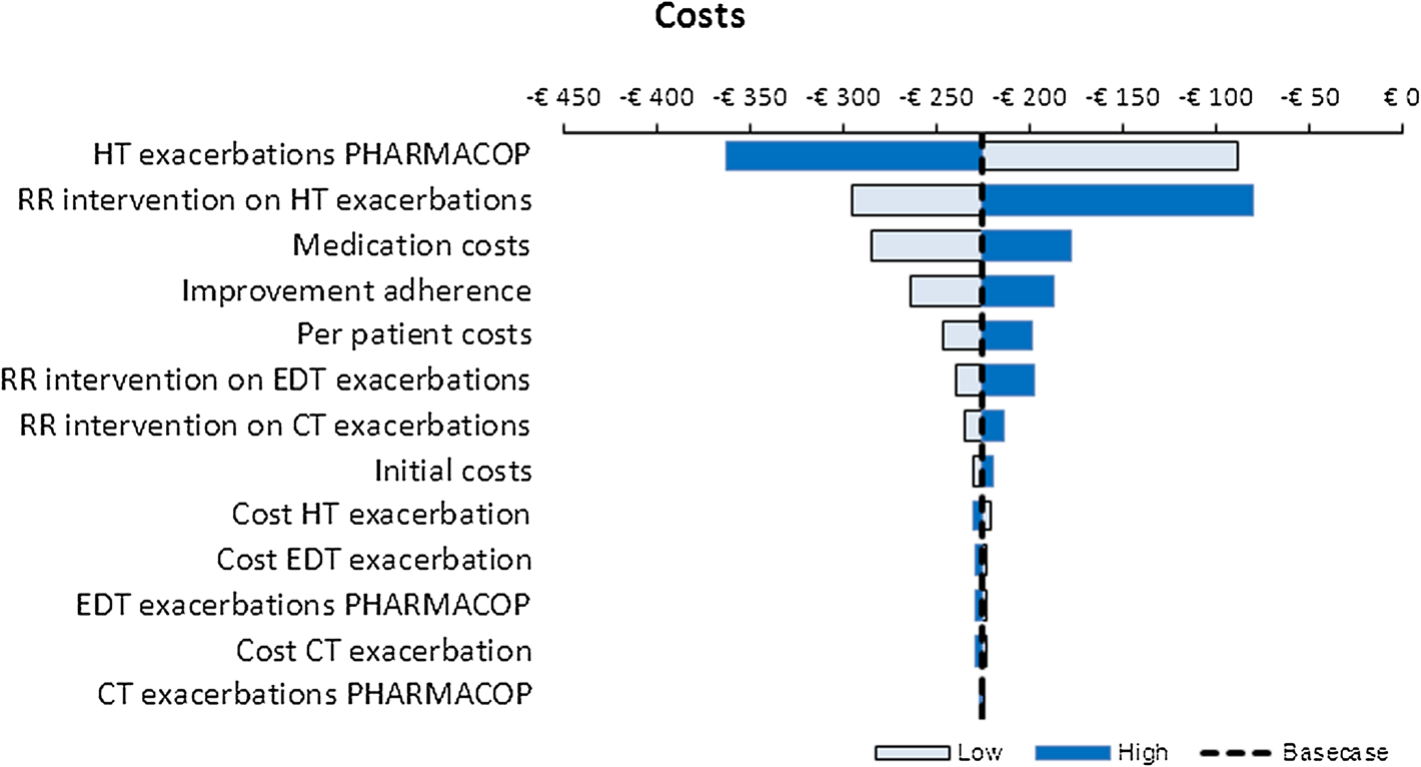
**Univariate sensitivity analyses.** CT: Community Treated; EDT: Emergency Department Treated; HT: Hospital Treated; RR: Relative Risk.

Probabilistic sensitivity analyses revealed that >99% of the 3,000 simulations performed resulted in cost-savings for the PHARMACOP-intervention, often combined with positive incremental effects on both QALYs and hospital-treated exacerbations. This is illustrated in Figures 4 and 5: The majority of the simulations were situated in the South-Eastern quadrant of the cost-effectiveness plane. At a willingness to pay of €0 per QALY, the probability of the PHARMACOP-intervention being cost-effective was 99.4%.In univariate sensitivity analyses, all relevant parameters were varied within their 95%CI of the basecase values. Figure 6 shows the model was most sensitive to the number of hospital-treated exacerbations in the PHARMACOP-trial and the relative risk reduction due to the intervention. The medication costs and adherence improvement were of somewhat less influence. However, the dominant situation of the PHARMACOP-intervention was retained in all univariate analyses.

In scenario analyses (Table 2) several variations of the intervention runtime, the time the adherence improvement would last, extensions of the time horizon and mean FEV_1_%pred were tested for their influence on cost-effectiveness. As no marked QALY differences were observed, this scenario analyses included costs and hospital-treated exacerbations only.

**Table 2:** Scenario analyses

**Effects on exacerbations last (years)**	**Time horizon (years)**	**Hospital-treated exacerbations**		
		**Usual care**	**PHARMACOP**	**Prevented**
*Basecase scenario (effects last for 3 months)*				
0.25	1	0.24	0.18	0.07
*Different time horizon*				
0.25	0.25	0.10	0.02	0.07
0.25	0.5	0.14	0.07	0.07
0.25	2	0.42	0.35	0.07
0.25	5	0.93	0.86	0.07
0.25	10	1.60	1.53	0.07
0.25	12.5	1.82	1.75	0.07
*Different program run time (effects linear extrapolated)*				
1	1	0.24	0.06	0.18
5	5	0.93	0.23	0.70
12.5	12.5	1.82	0.46	1.36
*Effect of adherence improvement lasts 2 years*				
0.25	5	0.93	0.86	0.07
*Effect of adherence improvement lasts 12.5 years*				
0.25	12.5	1.82	1.75	0.07
*Mean FEV*_*1*_*% pred of 40*				
0.25	1	0.25	0.18	0.07
0.25	5	1.02	0.95	0.07
0.25	10	1.75	1.68	0.07
*Mean FEV*_*1*_*% pred of 60*				
0.25	1	0.22	0.15	0.07
0.25	5	0.84	0.77	0.07
0.25	10	1.44	1.37	0.07
*Lower 95% CI RR effect on hospital treated exacerbations (RR = 0.121)*				
0.25	1	0.24	0.15	0.08
*Upper 95% CI RR effect on hospital treated exacerbations (RR = 0.523)*				
0.25	1	0.24	0.19	0.05

The PHARMACOP-intervention remained cost-saving with longer projected time horizons and different assumptions on the lasting effect on adherence. If the program runtime was as long as the time horizon, up to 1.36 hospital-treated exacerbations per patient were prevented in the 12.5 year time horizon. Cost savings were retained in most sensitivity analyses, except for the scenario where costs due to adherence improvement lasted for 12.5 years. Although the mean FEV_1_% pred did affect absolute number of hospital-treated exacerbations, the number of prevented hospital-treated exacerbations remained the same.

## Discussion

Our aim was to assess the cost-effectiveness of the PHARMACOP community pharmacists’ COPD intervention program. In a 1-year time horizon, the PHARMACOP-intervention would induce a cost saving of €227 per patient, compared to usual care. This was primarily the result of the prevention of 0.07 hospital-treated exacerbations per patient. Therefore, the results of this cost-effectiveness analysis indicate that the PHARMACOP-intervention provides more value for money, combined with increased health gains when compared to usual care, i.e. it is the dominant strategy. Furthermore, long-term projections revealed that when the intervention would be extended to longer periods (up to 12.5 years), a considerable amount of 1.36 hospital-treated exacerbations per patient would be prevented. As we assumed a linear extrapolation of effects, long-term clinical follow-up is necessary to confirm whether this assumption does reflect real-life effectiveness.

Accordingly, *Khdour et al.* reported a pharmacist’s intervention as highly cost-effective with both savings on total costs and gains in effects [32]. *Takemura et al.* did not report costs but observed comparable effects on adherence and exacerbation rates [33]. Notably, effects of these adherence enhancing interventions are considerable, especially when compared to reduction of exacerbations observed in trials assessing the effectiveness of (new) medication. This may be explained by the possibility that these type of behavior modifying interventions not just alter patients medication adherence but alter healthy behavior as a whole (i.e. “healthy adherer effect” [34]), resulting in a much larger effect. A posthoc analysis from the TORCH-trial [35] illustrates this explanation: patients with high adherence (regardless of whether the patients used medication or placebo) showed marked better outcomes (rate ratio severe exacerbations: 0.58) compared to patients with lower adherence. Moreover, regarding the percentage of patients with >1 exacerbations in the previous year, this was only 33% in the TORCH-trial, while in PHARMACOP this was 54%. As the ECLIPSE-study showed that a higher number of exacerbations in the previous year indicated a higher baseline risk for new exacerbations [36], more exacerbations could be expected in the PHARMACOP-population, thus larger potential gains. This aligns with the explanation regarding differences in effectiveness of COPD self-management interventions of which some showed positive and some showed disappointing results, depending on the baseline characteristics of the population [37]. While the PHARMACOP-intervention prevented a considerable amount of hospital-treated exacerbations, only little gains on quality of life measures were observed. This as well corresponds with results from a COPD intervention program that focused on adherence [9]. This finding is explained by the discrepancy between the timing of measurement of health status within trials and the relatively short duration of exacerbations [20].

When comparing the hospital-treated exacerbation rate (0.38 per patient-year) of the PHARMACOP-population to large trials, the rate seems relatively high. For example, in the TORCH-trial the hospitalization rate was 0.2 (SD:0.6) per year [38] and in the UPLIFT-trial 0.15 (SD 0.01) [39]. However, hospitalization rates highly depend on type of study and are reported to vary from as low as 0.09 to 2.4 per year [40]. Since co-morbidities such as heart failure increase the risk of hospital treated exacerbations in patients with COPD, and since several co-morbidities are excluded in classical RCTs of COPD (such as TORCH and UPLIFT), the different prevalences of co-morbidities might (partially) explain the higher exacerbation rate in the real-world PHARMACOP-study. Another risk factor for a high exacerbation rate was the winter season in which the trial was performed [41]. In addition, mean CAT score in the PHARMACOP-population was >10, indicating patients with high symptoms according to the new GOLD guidelines [4]. When compared to a previous Belgian cost-effectiveness report in the evaluation of tiotropium, hospitalization rates are more in line (0.36 per year) [25], what might suggest that patients in Belgium are relatively earlier referred to hospital when exacerbations are suspected.

### Strengths

This study is the first cost-effectiveness analysis of an intervention directed at improving COPD patients’ medication adherence and inhalation technique based on a large RCT. One of the major strengths of this trial was the community care setting in which real-life data were obtained. The vast majority of patients with COPD fill their prescriptions in community pharmacies, where recruitment took place. Our study population - and therefore also the results from the current cost-effectiveness study - is considered representative for the Belgian COPD population using inhaled medication for the maintenance treatment of COPD. The study closely followed and modeled real-life medication distributions among the study population. Using this distribution enabled precise estimates of the economic impact of improving medication adherence for the total population. Therefore, not only the costs for the intervention itself (materials and time of healthcare providers) but also costs related to the extent of medication use, which effects may last for longer periods, were included providing a complete economic picture. Finally, the analysis was reported according to the CHEERS-guidelines for reporting of health-economic evaluations [42].

### Limitations

Though basecase assumptions were well-considered and assessed for robustness in sensitivity analyses, long-term effects related to the programs’ future impact on frequency of (severe) exacerbations and costs, remain highly uncertain. Some studies recommend that inhalation instruction should be (frequently) reinforced for continuation of optimal pharmacological effects [43,44]. Also future changes in healthcare policies and treatments may change current cost-effectiveness estimates. Regarding implementation in the Belgian healthcare system, the varying ability of individual healthcare providers to adopt - and patients to respond to - the interventions has to be considered, which limits generalizability. Regarding generalizability to other countries, differences in country specific healthcare systems, costs and regulations should be taken into account. For example, this study did not take into account indirect costs (productivity), in line with Belgian guidelines, while the Dutch guidelines recommend to take these costs into account indeed [45].

Long-term projections of our COPD model should be interpreted with caution as e.g. in spite of evidence that decline in lung function is increased by the occurrence of an exacerbation we did not account for this possibility [46]. However, because this decline due to an exacerbation is relatively low in comparison with regular annual lung function decline, for analyses up to 2 years (including the basecase analyses) this is considered only a minor limitation.

### Recommendations

As an alternative to addition of new drugs to COPD patients’ treatment regimen, optimization of current treatment has to be considered. Pharmaceutical care (i.e. optimization of medication adherence and inhalation technique) as provided by the PHARMACOP-protocol should be embedded in the integral multidisciplinary respiratory care for patients with COPD. Based on the cost-saving strategy, health insurance companies should be stimulated to reimburse these type of interventions. Furthermore, community pharmacists are well positioned - and are recommended - to integrate COPD specific pharmaceutical care as part of their daily practices. Overall, these recommendations are expected to contribute to better patients outcomes and to lower total healthcare costs for the COPD population. In particular, when interventions are performed in the winter season, the season when patients are at highest risk for exacerbations, potential health and cost gains are maximized.

## Conclusions

In the current cost-effectiveness study of the PHARMACOP-trial, we demonstrate that improving inhaler adherence in community pharmacies is a cost-saving strategy compared with usual care. Before adding new therapies, the optimization of current treatment options has to be considered. Community pharmacies offer a cost-effective platform for improving medication adherence, inhalation technique and outcomes in patients with COPD and these activities should be embedded in the integral multidisciplinary respiratory care for patients with COPD.

## Competing interests

JB, ET, KB, EM, SV and GB declare that they have no competing interests, regarding the submitted work. MP reports grants, personal fees and non-financial support from various pharmaceutical companies, outside the submitted work. The Erasmus University, Institute for Medical Technology Assessment, where MR is employed, has received funding for designing and conducting cost-effectiveness studies of COPD drugs from multiple pharmaceutical companies (Boehringer Ingelheim, Nycomed, Pfizer). MR has received speaker fees and compensation for serving on advisory boards for GSK, Boehringer Ingelheim, Pfizer, Nycomed and Novartis. MR does not own stock of any pharmaceutical company.

## Authors’ contributions

JB, SV, MR and MP designed the research. JB and SV performed the analyses. ET, KB, EM, MR and GB provided input for the analyses. All authors interpreted the data. JB and ET wrote the paper. All authors commented on the first draft. All authors read and approved the final version of the manuscript.

## Supplementary Material

Additional file 1 **Summary of the PHARMACOP study [**[11]**,**[47]**].** Click here for file

Additional file 2 Comparison of model results after three months to PHARMACOP RCT results. Click here for file

## References

[B1] World Health Organization Chronic obstructive pulmonary disease http://www.who.int/respiratory/copd/en

[B2] van Boven JF Vegter S van der Molen T Postma MJ COPD in the working age population: the economic impact on both patients and government COPD 2013 10 6 629 639 10.3109/15412555.2013.813446 23845002

[B3] Scientific Institute for Public Health Belgium (WIV-ISP) https://www.wiv-isp.be

[B4] From the Global Strategy for the Diagnosis, Management and Prevention of COPD, Global Initiative for Chronic Obstructive Lung Disease (GOLD) 2013 http://www.goldcopd.org

[B5] Mehuys E Boussery K Adriaens E Van Bortel L De Bolle L Van Tongelen I Remon JP Brusselle G COPD management in primary care: an observational, community pharmacy-based study Ann Pharmacother 2010 44 2 257 266 10.1345/aph.1M481 20103611

[B6] Melani AS Bonavia M Cilenti V Cinti C Lodi M Martucci P Serra M Scichilone N Sestini P Aliani M Neri M Gruppo Educazionale Associazione Italiana Pneumologi Ospedalieri Inhaler mishandling remains common in real life and is associated with reduced disease control Respir Med 2011 105 6 930 938 10.1016/j.rmed.2011.01.005 21367593

[B7] van Boven JF Chavannes NH van der Molen T Rutten-van Molken MP Postma MJ Vegter S Clinical and economic impact of non-adherence in COPD: a systematic review Respir Med 2014 108 1 103 113 10.1016/j.rmed.2013.08.044 24070566

[B8] Casas A Troosters T Garcia-Aymerich J Roca J Hernandez C Alonso A del Pozo F de Toledo P Anto JM Rodriguez-Roisin R Decramer M Members of the CHRONIC Project Integrated care prevents hospitalisations for exacerbations in COPD patients Eur Respir J 2006 28 1 123 130 10.1183/09031936.06.00063205 16611656

[B9] Takemura M Mitsui K Ido M Matsumoto M Koyama M Inoue D Takamatsu K Itotani R Ishitoko M Suzuki S Aihara K Sakuramoto M Kagioka H Fukui M Effect of a network system for providing proper inhalation technique by community pharmacists on clinical outcomes in COPD patients Int J Chron Obstruct Pulmon Dis 2013 8 239 244 23696699 10.2147/COPD.S44022 PMC3656647

[B10] Jarab AS Alqudah SG Khdour M Shamssain M Mukattash TL Impact of pharmaceutical care on health outcomes in patients with COPD Int J Clin Pharm 2012 34 1 53 62 10.1007/s11096-011-9585-z 22101426

[B11] Tommelein E Mehuys E Van Hees T Adriaens E Van Bortel L Christiaens T Van Tongelen I Remon JP Boussery K Brusselle G Effectiveness of pharmaceutical care for patients with chronic obstructive pulmonary disease (PHARMACOP): a randomized controlled trial Br J Clin Pharmacol 2014 77 5 756 766 10.1111/bcp.12242 24117908 PMC4004396

[B12] Hoogendoorn M van Wetering CR Schols AM Rutten-van Molken MP Is INTERdisciplinary COMmunity-based COPD management (INTERCOM) cost-effective? Eur Respir J 2010 35 1 79 87 10.1183/09031936.00043309 19574331

[B13] Steuten LM Lemmens KM Nieboer AP Vrijhoef HJ Identifying potentially cost effective chronic care programs for people with COPD Int J Chron Obstruct Pulmon Dis 2009 4 87 100 19436687 PMC2672791

[B14] Boland MR Tsiachristas A Kruis AL Chavannes NH Rutten-van Molken MP The health economic impact of disease management programs for COPD: a systematic literature review and meta-analysis BMC Pulm Med 2013 13 40 2466-13-40 10.1186/1471-2466-13-40 23819836 PMC3704961

[B15] Bryant J McDonald VM Boyes A Sanson-Fisher R Paul C Melville J Improving medication adherence in chronic obstructive pulmonary disease: a systematic review Respir Res 2013 14 1 109 10.1186/1465-9921-14-109 24138097 PMC4015036

[B16] Hess LM Raebel MA Conner DA Malone DC Measurement of adherence in pharmacy administrative databases: a proposal for standard definitions and preferred measures Ann Pharmacother 2006 40 7–8 1280 1288 16868217 10.1345/aph.1H018

[B17] Menn P Leidl R Holle R A lifetime Markov model for the economic evaluation of chronic obstructive pulmonary disease Pharmacoeconomics 2012 30 9 825 840 10.2165/11591340-000000000-00000 22799876

[B18] Oostenbrink JB Rutten-van Molken MP Monz BU FitzGerald JM Probabilistic Markov model to assess the cost-effectiveness of bronchodilator therapy in COPD patients in different countries Value Health 2005 8 1 32 46 10.1111/j.1524-4733.2005.03086.x 15841892

[B19] Starkie HJ Briggs AH Chambers MG Pharmacoeconomics in COPD: lessons for the future Int J Chron Obstruct Pulmon Dis 2008 3 1 71 88 18488430 PMC2528220

[B20] Rutten-van Molken MP Goossens LM Cost effectiveness of pharmacological maintenance treatment for chronic obstructive pulmonary disease: a review of the evidence and methodological issues Pharmacoeconomics 2012 30 4 271 302 10.2165/11589270-000000000-00000 22409290

[B21] Cleemput I Crott R Vrijens F Huybrechts M Van Wilder P Ramaekers D Voorlopige richtlijnen voor farmaco-economisch onderzoek in België Health Technology Assessment (HTA) 2006 Brussels: Belgian Health Care Knowledge Centre (KCE). KCE reports 28A D2006/10.273/10

[B22] Hoogendoorn M Feenstra TL Hoogenveen RT Al M Molken MR Association between lung function and exacerbation frequency in patients with COPD Int J Chron Obstruct Pulmon Dis 2010 5 435 444 21191438 10.2147/COPD.S13826 PMC3008329

[B23] Belgisch Centrum voor Farmacotherapeutische Informatie (B.C.F.I. VZW) http://www.bcfi.be

[B24] Oostenbrink JB Rutten-van Molken MP Resource use and risk factors in high-cost exacerbations of COPD Respir Med 2004 98 9 883 891 10.1016/j.rmed.2004.02.013 15338802

[B25] Neyt M Van den Bruel A Gailly J Thiry N Devriese S Tiotropium in the Treatment of Chronic Obstructive Pulmonary Disease: Health Technology Assessment Health Technology Assessment (HTA) 2009 Brussels: Belgian Health Care Knowledge Centre (KCE). KCE reports 108C D/2009/10.273/20

[B26] Borg S Ericsson A Wedzicha J Gulsvik A Lundback B Donaldson GC Sullivan SD A computer simulation model of the natural history and economic impact of chronic obstructive pulmonary disease Value Health 2004 7 2 153 167 10.1111/j.1524-4733.2004.72318.x 15164805

[B27] Hoogendoorn M Rutten-van Molken MP Hoogenveen RT Al MJ Feenstra TL Developing and applying a stochastic dynamic population model for chronic obstructive pulmonary disease Value Health 2011 14 8 1039 1047 10.1016/j.jval.2011.06.008 22152172

[B28] Hoogendoorn M Rutten-van Molken MP Hoogenveen RT van Genugten ML Buist AS Wouters EF Feenstra TL A dynamic population model of disease progression in COPD Eur Respir J 2005 26 2 223 233 10.1183/09031936.05.00122004 16055869

[B29] Scanlon PD Connett JE Waller LA Altose MD Bailey WC Buist AS Smoking cessation and lung function in mild-to-moderate chronic obstructive pulmonary disease. the lung health study Am J Respir Crit Care Med 2000 161 2 Pt 1 381 390 10673175 10.1164/ajrccm.161.2.9901044

[B30] Wysocki T Greco P Harris MA Bubb J White NH Behavior therapy for families of adolescents with diabetes: maintenance of treatment effects Diabetes Care 2001 24 3 441 446 10.2337/diacare.24.3.441 11289465

[B31] Briggs AH Claxton K Sculpher MJ Decision modelling for Health Economic Evaluation 2006 New York: Oxford University Press

[B32] Khdour MR Agus AM Kidney JC Smyth BM McElnay JC Crealey GE Cost-utility analysis of a pharmacy-led self-management programme for patients with COPD Int J Clin Pharm 2011 33 4 665 673 10.1007/s11096-011-9524-z 21643784

[B33] Takemura M Mitsui K Itotani R Ishitoko M Suzuki S Matsumoto M Aihara K Oguma T Ueda T Kagioka H Fukui M Relationships between repeated instruction on inhalation therapy, medication adherence, and health status in chronic obstructive pulmonary disease Int J Chron Obstruct Pulmon Dis 2011 6 97 104 21407822 10.2147/COPD.S16173 PMC3048085

[B34] Simpson SH Eurich DT Majumdar SR Padwal RS Tsuyuki RT Varney J Johnson JA A meta-analysis of the association between adherence to drug therapy and mortality BMJ 2006 333 7557 15 10.1136/bmj.38875.675486.55 16790458 PMC1488752

[B35] Vestbo J Anderson JA Calverley PM Celli B Ferguson GT Jenkins C Knobil K Willits LR Yates JC Jones PW Adherence to inhaled therapy, mortality and hospital admission in COPD Thorax 2009 64 11 939 943 10.1136/thx.2009.113662 19703830

[B36] Hurst JR Vestbo J Anzueto A Locantore N Mullerova H Tal-Singer R Miller B Lomas DA Agusti A Macnee W Calverley P Rennard S Wouters EF Wedzicha JA Evaluation of COPD Longitudinally to Identify Predictive Surrogate Endpoints (ECLIPSE) Investigators: Susceptibility to exacerbation in chronic obstructive pulmonary disease N Engl J Med 2010 363 12 1128 1138 10.1056/NEJMoa0909883 20843247

[B37] Bourbeau J Not all self-management programs in chronic obstructive pulmonary disease have positive results: why is replication a problem? Chron Respir Dis 2004 1 1 5 6 16281662 10.1191/1479972304cd006ed

[B38] Calverley PM Anderson JA Celli B Ferguson GT Jenkins C Jones PW Yates JC Vestbo J TORCH investigators Salmeterol and fluticasone propionate and survival in chronic obstructive pulmonary disease N Engl J Med 2007 356 8 775 789 10.1056/NEJMoa063070 17314337

[B39] Tashkin DP Celli B Senn S Burkhart D Kesten S Menjoge S Decramer M UPLIFT Study Investigators A 4-year trial of tiotropium in chronic obstructive pulmonary disease N Engl J Med 2008 359 15 1543 1554 10.1056/NEJMoa0805800 18836213

[B40] Seemungal TA Hurst JR Wedzicha JA Exacerbation rate, health status and mortality in COPD–a review of potential interventions Int J Chron Obstruct Pulmon Dis 2009 4 203 223 19554195 10.2147/copd.s3385 PMC2699821

[B41] Jenkins CR Celli B Anderson JA Ferguson GT Jones PW Vestbo J Yates JC Calverley PM Seasonality and determinants of moderate and severe COPD exacerbations in the TORCH study Eur Respir J 2012 39 1 38 45 10.1183/09031936.00194610 21737561

[B42] Husereau D Drummond M Petrou S Carswell C Moher D Greenberg D Augustovski F Briggs AH Mauskopf J Loder E ISPOR Health Economic Evaluation Publication Guidelines-CHEERS Good Reporting Practices Task Force Consolidated Health Economic Evaluation Reporting Standards (CHEERS)–explanation and elaboration: a report of the ISPOR Health Economic Evaluation Publication Guidelines Good Reporting Practices Task Force Value Health 2013 16 2 231 250 10.1016/j.jval.2013.02.002 23538175

[B43] Crompton GK Barnes PJ Broeders M Corrigan C Corbetta L Dekhuijzen R Dubus JC Magnan A Massone F Sanchis J Viejo JL Voshaar T Aerosol Drug Management Improvement Team The need to improve inhalation technique in Europe: a report from the Aerosol Drug Management Improvement Team Respir Med 2006 100 9 1479 1494 10.1016/j.rmed.2006.01.008 16495040

[B44] Lavorini F Magnan A Dubus JC Voshaar T Corbetta L Broeders M Dekhuijzen R Sanchis J Viejo JL Barnes P Corrigan C Levy M Crompton GK Effect of incorrect use of dry powder inhalers on management of patients with asthma and COPD Respir Med 2008 102 4 593 604 10.1016/j.rmed.2007.11.003 18083019

[B45] Health Care Insurance Board Dutch pharmacoeconomic guidelines [in Dutch] http://www.zorginstituutnederland.nl/binaries/content/documents/zinl-www/documenten/publicaties/publications-in-english/2006/0604-guidelines-for-pharmacoeconomic-research/0604-guidelines-for-pharmacoeconomic-research/Guidelines+for+pharmacoeconomic+research.pdf

[B46] Makris D Moschandreas J Damianaki A Ntaoukakis E Siafakas NM Milic Emili J Tzanakis N Exacerbations and lung function decline in COPD: new insights in current and ex-smokers Respir Med 2007 101 6 1305 1312 10.1016/j.rmed.2006.10.012 17112715

[B47] NICE Clinical Guideline 76: Medicines adherence 2009 National Institute for Health and Clinical Excellence http://www.nice.org.uk/nicemedia/live/11766/43042/43042.pdf

