# Supplementary material for: Improving inhaler adherence in patients with Chronic Obstructive Pulmonary Disease: a cost-effectiveness analysis
Source: Respir Res. 2014 Jun 14;15(1):66. doi: 10.1186/1465-9921-15-66 (PMC4067522; doi:10.1186/1465-9921-15-66)
Supplement: Additional file 1 — Summary of the PHARMACOP study [[11],[47]]. [file 1465-9921-15-66-S1.docx]

Additional file 1: Summary of the PHARMACOP study

Study protocol

The PHARMACOP study was a 3-month randomized controlled trial (N = 734) carried out between December 2010 and July 2011 in 170 community pharmacies throughout Belgium. Before start of the trial, all participating pharmacists received a training session addressing COPD (non) pharmacological management, referral criteria and the study protocol. To support interventions, pharmacists were provided with patient information leaflets, demo inhalers and a list of practical solutions to tackle specific non-adherent behavior [47].

Patients in the intervention group received interventions using one-on-one counseling sessions at study start and after 1 month of follow-up. Interventions included verbal and written information about COPD pathophysiology, COPD medication (dose and time of intake, inhalation technique, importance of adherence and side effects) and self-management (e.g. lifestyle advise, smoking cessation). Control patients were given usual care (e.g. recommendations on dose and time of intake only). Duration of counseling sessions was estimated to be 15 to 25 minutes according to patients’ needs. Patients were included in the PHARMACOP study if they met the following inclusion criteria: prescription for inhaled COPD maintenance medication, aged 50 years and older and a smoking history of at least 10 pack-years.

PHARMACOP population

In the PHARMACOP population 66% were male, mean age at baseline was 68.7 years (SD: 9.6), 43% were current smokers and mean COPD duration was 11 years (SD: 9). Regarding health status and symptoms: 23% had modified Medical Research Council (mMRC) scores of 3 or 4, mean COPD Assessment Test (CAT) score was 16.5 (SD: 7.7) and 54% had 1 or more exacerbations in the preceding year.

Summary of results

The PHARMACOP trial improved inhalation scores with 13.5% (95%CI: 10.8-16.1; P < 0.0001). Medication adherence, as measured by proportion of days covered, was improved from 85.70% to 94.21% (difference: 8.51%, 95%CI: 4.63-12.4; P < 0.0001). In the intervention group a significantly lower hospitalization rate was observed (9 vs 35; Rate ratio: 0.28, 95%CI: 0.12-0.64; P = 0.003). No other significant differences were observed. A complete description of the PHARMACOP study is provided elsewhere [11].
