# Supplementary material for: Improving inhaler adherence in patients with Chronic Obstructive Pulmonary Disease: a cost-effectiveness analysis
Source: Respir Res. 2014 Jun 14;15(1):66. doi: 10.1186/1465-9921-15-66 (PMC4067522; doi:10.1186/1465-9921-15-66)
Supplement: Additional file 2 — Comparison of model results after three months to PHARMACOP RCT results. [file 1465-9921-15-66-S2.docx]

Additional file 2: Comparison of model results after three months to PHARMACOP RCT results

Simulation

In Table 3 the model results of a cohort (N = 363) that was followed for the first three months are compared to the real results from the PHARMACOP-trial. Results did not differ significantly (P > 0.05).

**Table 3** Comparison of the number of exacerbations between the model and PHARMACOP trial (3-month time-horizon)

|  | Usual care | | PHARMACOP | | Difference (95%CI) | |
| --- | --- | --- | --- | --- | --- | --- |
|  | RCT | Model | RCT | Model | RCT | Model |
| Cohort | 363 | 363 | 371 | 363 | - | - |
| Community treated exacerbations | 194 | 191 | 179 | 173 | −15 (−44.8; 7.4) | −18 (−50.1; 20.3) |
| Hospital treated exacerbations | 35 | 34 | 9 | 9 | −26 (−39.5; −13.9) | −25 (−35.5; −14.4) |
| ED treated exacerbations | 18 | 17 | 15 | 15 | −3 (−7.9; 14.8) | −2 (−11.1; 11.5) |

ED: Emergency Department; RCT: Randomized Controlled Trial.
